# Dietary β-hydroxy-β-methyl butyrate supplementation improves intestinal health and growth performance in Tibetan sheep lambs via modulating small intestinal microbiota

**DOI:** 10.1186/s40104-025-01345-z

**Published:** 2026-02-09

**Authors:** Jieqiong Cai, Weibin Wu, Lamei Wang, Dandan Meng, Hao Yang, Shimin Liu, Shengzhen Hou, Yangchun Cao

**Affiliations:** 1 College of Animal Science and Technology, Northwest A&F University, Xianyang, 712100 China; 2 College of Agriculture and Animal Husbandry, Qinghai University, Xining, 810016 China; 3 Key Laboratory of Qinghai-Tibet Plateau Grazing Yak and Tibetan Sheep Animal Nutrition and Feed-Forage, Ministry of Agriculture and Rural Affairs, State Key Laboratory of Plateau Ecology and Agriculture, Qinghai University, Xining, 810016 China; 4 UWA Institute of Agriculture, The University of Western Australia, Crawley, WA 6009 Australia

**Keywords:** Antioxidant capacity, Carbohydrate metabolism, Metagenomics, Microbial composition, Short-chain fatty acids

## Abstract

**Background:**

Tibetan sheep grazing on the Qinghai-Tibet Plateau require dietary protein supplementation; however, they face economic constraints due to the high cost of feed transportation in this region. Given that the leucine metabolite β-hydroxy-β-methyl butyrate (HMB) enhances both protein synthesis and intestinal nutrient absorption, this study employed metagenomics and untargeted metabolomics to systematically evaluate HMB's effects on antioxidant capacity, immune response, microbiota, metabolites, and the health of the small intestine in Tibetan sheep. A total of 120 healthy weaned 60-day-old male Tibetan lambs were assigned to diets containing 0 mg/kg (control group, CON), 430 mg/kg (low HMB, L-HMB), 715 mg/kg (medium HMB, M-HMB), or 1,000 mg/kg (high HMB, H-HMB) for 90 d. At the end of the experiment, 6 lambs from each group were slaughtered for intestinal tissue and content sampling.

**Results:**

The M-HMB treatment significantly increased average daily gain of the lambs without affecting feed intake, thereby improving feed utilization efficiency. M-HMB promoted the development of small intestinal morphological and elevated villus height, while also enhancing the activities of digestive enzyme and disaccharidase activities. Furthermore, M-HMB enhanced the antioxidant capacity, immune response, and barrier function of the small intestine. Metagenomic analysis revealed that M-HMB supplementation improved the composition of the small intestinal microbiota in Tibetan sheep, specifically increasing the relative abundance of *Ruminococcus bacterium P7* and *R. bromii*, and enhanced microbial carbohydrate degradation capacity. Metabolomic analysis demonstrated that M-HMB supplementation significantly altered the small intestinal metabolite profile, enhancing carbohydrate metabolic pathways and increased the production of short-chain fatty acids (SCFAs). M-HMB upregulated PLCβ1 and ERK1/2 protein expression levels in small intestinal tissue and elevated the proportion of Ki67-positive cells at the basal crypt region of small intestinal crypts, suggesting enhanced proliferative activity of intestinal epithelial cells.

**Conclusions:**

In summary, dietary supplementation with M-HMB (715 mg/kg) promoted small intestinal growth and development, enhanced digestive and absorptive functions, optimized the microbial composition, improved carbohydrate degradation, and increased the production of SCFAs, ultimately improving the growth performance of Tibetan sheep lambs.

**Graphical Abstract:**

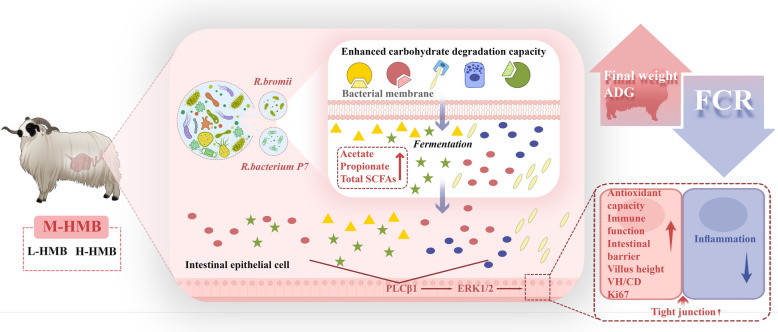

**Supplementary Information:**

The online version contains supplementary material available at 10.1186/s40104-025-01345-z.

## Introduction

As a key livestock species on the Qinghai-Tibet Plateau, Tibetan sheep exhibit unique ecological adaptations to high-altitude hypoxia, making them indispensable for regional pastoral systems. Currently, herders typically begin supplementing Tibetan lambs with high-protein feed at two months of age to ensure sufficient body weight by six months, thereby improving market readiness [1]. However, this early nutritional regime, combined with the Plateau's harsh environment and high feed transportation costs, significantly reduces the economic returns for herders.

β-Hydroxyl-β-methyl butyrate (HMB), a natural metabolite of leucine, stimulates protein synthesis at much lower doses (3 g HMB is equivalent to 60 g leucine), promotes anticatabolic process, and supports tissue repair under pathological or stress conditions. HMB also counteracts intestinal injury and improves intestinal functions [2–4]. Previous studies have demonstrated that HMB promotes intestinal epithelial cell differentiation and maturation in broilers and enhances abdominal fat deposition by modulating gut microbiota function in laying hens [5, 6]. In weaning piglets, HMB improves intestinal barrier function and preserves intestinal integrity under lipopolysaccharide challenge [7, 8]. However, the effects of HMB on growth performance and intestinal health in Tibetan sheep have not been thoroughly investigated.

The small intestine is the primary site of post ruminal digestion and absorption in ruminants. Its energy efficiency in starch digestion and absorption surpasses that of ruminal fermentation [9]. Due to the dominant role of the rumen in the ruminant digestive system, the development and functions of the small intestine are often overlooked, despite its critical roles in nutrient digestion and absorption, mucosal immune regulation, and overall growth and development in young ruminants. It also contains the largest immune mass in the body and its mucosa is densely populated with T and B lymphocytes, plasma cells, mast cells, macrophages, and intraepithelial lymphocytes [10]. There are strong interactions among intestinal epithelial cells, the immune components, and resident microbiota, forming a complex and highly dynamic intestinal micro-ecosystem. Alterations in the small intestinal microbiota can disturb these interactions, affecting nutrient digestion and absorption and immune responses [11, 12].

Therefore, we hypothesized that dietary HMB supplementation would modulate the intestinal microbiota and its interactions with the intestinal epithelium and immune components, resulting in improvements in nutrient digestion and absorption in the intestine and growth performance of young animals. To test this hypothesis, we used weaned Tibetan lambs as a model and employed metagenomic and untargeted metabolomic techniques to evaluate how dietary HMB supplementation influences these microbial-epithelial interactions, aiming for improving feed efficiency and economic sustainability of Tibetan sheep production.

## Materials and methods

### Ethical statement

The protocol and details of the present study were approved by the Institution of Animal Care and Use Committee of Northwest A&F University (DK2024016).

### Experimental design

The experiment was conducted at the Jinzang Ecological Plateau Tibetan Sheep Breeding Cooperative in Haiyan Count (100.99° E, 36.90° N), Haibei Prefecture, Qinghai Province, China. The altitude of the location is 3,256 m. The experiment began in March, coinciding with the spring season. A total of 120 healthy, weaned male Tibetan lambs, 60 days of age, were assigned to four groups (*n* = 30) with similar live weights, and randomly assigned to four treatments with HMB (HM20220828, TSI Group, Shanghai, China) supplementation of 0 mg/kg (CON), 430 mg/kg (L-HMB), 715 mg/kg (M-HMB), or 1,000 mg/kg (H-HMB). The M-HMB dosage was determined in our previous study as the optimal level to improve lipid metabolism in the subcutaneous fat in Tibetan sheep [13]. HMB was initially mixed with the premix, then mixed into the concentration, and finally mixed with the roughages to make a total mixed ration (TMR).

Animal management during the experiment followed our previously established protocols [14], Briefly, all lambs were individually penned in an animal house and had ad libitum access to feed and water. The basal diet was formulated to meet the nutrient requirements of growing lambs according to nutrient requirements of mutton sheep and goat (NY/T 816–2021) [15], and its gradient composition and nutrient concentrations are presented in Table 1. The diet was prepared as a TMR and fed to lambs twice daily at 07:00 and 13:00. Offered feed and residuals were recorded daily to calculated feed intake. The experiment lasted for 100 d, consisting of a 10-day adaptation period on the basal diet followed by 90 days of feeding the basal diet supplemented with the designated HMB levels.

**Table 1:** Composition and nutrient concentrations of the basal diet (dry matter basis), %

Ingredients	Content
Oat hey	15.00
Oat silage	15.00
Corn	36.05
Soybean meal	1.40
Rapeseed meal	8.96
Cottonseed meal	1.40
Palm meal	17.50
NaCl	0.70
Limestone	0.70
Baking soda	0.07
Premix^a^	3.22
Nutrient levels^b^	
Digestible energy, MJ/kg	9.58
Crude protein	11.83
Ether extract	3.58
Crude fiber	15.90
Neutral detergent fiber	36.99
Acid detergent fiber	25.68
Ca	0.99
P	0.57

^a^The premix provided per kilogram of TMR (dry matter basis): Cu 18 mg, Fe 66 mg, Zn 30 mg, Mn 48 mg, Se 0.36 mg, I 0.6 mg, Co 0.24 mg, VA 24,000 IU, VD 4,800 IU, VE 48 IU

^b^Digestible energy value was calculated while the other nutrients were the measured values

### Sample collection

At the end of the experiment, 24 Tibetan lambs (*n* = 6 from each group) were randomly selected and slaughtered after 16 h of fasting. Jejunal and ileal contents were collected and stored at −80 °C for subsequent analysis. Mid-segments of jejunal and ileal tissues were sampled and divided into two aliquots: 1) fixed in 4% paraformaldehyde for histological analysis, and 2) stored at −80 °C for subsequent analysis.

### Chemical analysis

Nutrient compositions of diets was analyzed according to the AOAC protocols [16]: dry matter (930.15), crude protein (984.13), ether extract (2003.06), crude fiber (978.10), neutral detergent fiber (2002.04), and acid detergent fiber (973.18). Calcium and phosphorus concentrations were determined using Inductively Coupled Plasma-Optical Emission Spectroscopy (ICP-OES) following AOAC method 985.01.

### Enzyme-linked immunosorbent assay

Antioxidant parameters (catalase, CAT; superoxide dismutase, SOD; glutathione peroxidase, GSH-Px; total antioxidant capacity, T-AOC; malondialdehyde, MDA) and immune indicators (interleukin-1 beta, IL-1β; IL-6; IL-8; IL-10; immunoglobulin A, IgA; IgG; IgM; tumor necrosis factor alpha, TNF-α) in jejunal and ileal tissues were measured using commercial ELISA kits (Jiangsu Meimian Industrial, Yancheng, China) according to the manufacturer's protocols. Digestive enzymes (α-amylase, chymotrypsin, cellulase, trypsin, and lipase) and disaccharidases (maltase, sucrase, and lactase) activities in jejunal and ileal contents were measured using commercial ELISA kits (Jiangsu Meimian Industrial, Yancheng, China).

### Jejunum and ileum morphology

The mid-segment jejunal and ileal tissue samples were fixed in 4% paraformaldehyde, paraffin-embedded, and sectioned. The sections were dewaxed in xylene, rehydrated through a graded ethanol series, and stained with hematoxylin and eosin. Five sections of each hematoxylin–eosin-stained sample were randomly selected and observed using BX51 microscope (Olympus, Japan). Villus height and crypt depth were quantified using ImageJ analysis software and the villi height to crypt depth ratio (VH/CD) was calculated. Tissue samples were further processed for ultrastructural analysis using SU8100 scanning electron microscopy (Hitachi, Japan) and HT7800 transmission electron microscopy (Hitachi, Japan) following the standard protocols.

### Immunofluorescence staining

Ileal tissue proliferate activity was assessed using a standard immunofluorescent staining protocol on paraffin-embedded ileal tissue. Briefly, sections were dehydrated and fixed, then incubated overnight at 4 °C with primary antibody Ki67 (GB111499, Servicebio, China). CY3-labeled secondary antibody was subsequently added and incubated at room temperature for 50 min. Sections were observed using an upright fluorescence microscope (NIKON, Japan) and quantified with ImageJ software (National Institutes of Health, USA).

### Metagenomic analysis of jejunal and ileal contents

Four content samples were randomly selected from the six slaughtered lambs in both CON and M-HMB groups. Total microbial DNA in jejunal and ileal content samples was extracted using HiPure Bacterial DNA kits (Magen, Guangzhou, China) according to the manufacturer’s instructions. The DNA purity and quantity were evaluated using NanoDrop (Thermo Fisher Scientific, USA) accordingly. Sequencing was performed on the Illumina Novaseq 6000 platform using paired-end technology (PE 150; Illumina, USA) at Genedenovo Biotechnology Co., Ltd. (Guangzhou, China). Raw data from Illumina platform were filtered using FASTP (version 0.18.0) by removing reads with ≥ 10% unidentified nucleotides (N), quality scores ≤ 20, and reads aligned to the barcode adapter. Reads were aligned and filtered out host sequences using Bowtie (version 2.2.5) to sheep (GCA_000298735.1). Clean reads of each sample were assembled individually using MEGAHIT (version 1.1.2) stepping over a k-mer range of 21 to 141 (or 27 to 127) to generate sample (or group)-derived assembly. Genes were predicted based on the final assembly contigs (> 500 bp) using MetaGeneMark (version 3.38). The predicted genes ≥ 300 bp in length from all samples were pooled and combined based on ≥ 95% identity and 90% reads coverage using CD-HIT (version 4.6) to reduce the number of redundant genes for the downstream assembly step. The unigenes were annotated using DIAMOND (version 0.9.24) by aligning with the deposited ones in diverse protein databases including National Center for Biotechnology Information non-redundant protein database and Kyoto Encyclopedia of Genes and Genomes (KEGG). Carbohydrate-active enzyme annotation was conducted basing on Carbohydrate-Active enZYmes Database (CAZy). Welch's *t*-test was used to assess the statistical significance of KEGG and CAZy pathway enrichment results. α-Diversity was calculated using Python scikit-bio (v0.5.6), and β-diversity using the R Vegan package. Linear discriminant analysis effect size (LEfSe) was performed to identify differentially abundant taxa. Taxa meeting both criteria, *P* < 0.05 and log_10_(LDA score) ≥ 2 in absolute value, were considered significant.

### Untargeted metabolomics in jejunal and ileal contents

Untargeted metabolomics in six jejunal and six ileal content samples from each CON and M-HMB groups were analyzed using UHPLC system (1290 Infinity LC, Agilent Technologies) coupled to a quadrupole time of flight (AB Sciex TripleTOF 6600) by Shanghai Applied Protein Technology Co., Ltd. For HILIC separation, a 2.1 mm × 100 mm ACQUITY UPLC BEH 1.7 μm column (Waters, Ireland) was used. The raw MS data were converted to MzXML files using ProteoWizard MSConvert before importing into XCMS software (https://proteowizard.sourceforge.io/download.html, accessed on 7 November 2023). CAMERA (Collection of Algorithms of MEtabolite pRofile Annotation) was sued for annotation of adducts. Briefly, after normalized to total peak intensity, the processed data were analyzed by R package ropls (Version 3.3.2) for partial least-squares discriminant analysis (PLS-DA). The differential metabolites were identified by *P* value < 0.01, variable importance projection (VIP) value > 1, and FC > 1.5 or < 0.67. The differential expressed metabolites were also mapped to the KEGG pathway database.

### Short chain fatty acids in jejunal and ileal contents

The concentrations of short chain fatty acids (SCFAs) in the jejunal and ileal content samples were detected by gas chromatography–mass spectrometry (7890B GC System, Aglient, Billerica, MA, USA) with an Agilent DB-FFAP capillary column (30 m × 250 µm × 0.25 µm). The column temperature was programmed as follows: initial 90 °C held for 1 min, ramp to 160 °C at 10 °C/min and held for 5 min, then further to 220 °C at 10 °C/min and held for 15 min. Helium was used as the carrier gas at 1.0 mL/min.

### Quantitative real-time qRT-PCR analysis

Total RNA in jejunal and ileal tissues was extracted using the TRIzol method. RNA purity and integrity were confirmed (A_260_/A_230_ and A_260_/A_280_ between 1.80–2.20). Reverse transcription was performed using the Prime Script™ RT Kit (Takara, Japan) and qPCR was performed using the Hieff qPCR SYBR Green Master Mix (Yeasen Biotechnology, Shanghai, China) on a QuantStudio 5 real-time PCR instrument (Applied Biosystems, California, USA). The relative expression of the gene was calculated using the 2^−∆∆Ct^ method. The β-actin gene was used as the reference. Information on the primer sequences are shown in Table S1.

### Western blotting

Proteins in three ileal tissue samples randomly selected from the six slaughtered lambs from each Con and M-HMB groups were extracted using RIPA lysis buffer (P0013B, Beyotime, China), with concentrations quantified in BCA assay (BL521S, Biosharp, China) and normalized for equal loading. Following SDS-PAGE separation, proteins were transferred to PVDF membranes, blocked with 5% BSA for 2 h, and probed with primary antibodies at 4 °C overnight. The primary antibodies used include PLCβ1 (AB_2838688, Affinity, USA), p-ERK1/2 (bs-3016R, Bioss, China), ERK1/2 (bs-2637R, Bioss, China), and GAPDH (60004-1-Ig, Proteintech, China). After washing, membranes were incubated with species-matched secondary antibodies, and target proteins were visualized by enhanced chemiluminescence. Band intensities were quantified using ImageJ software (National Institutes of Health, USA).

### Statistical analysis

Jejunal and ileal morphology, digestive enzyme activities, antioxidant parameters, SCFAs, and immune indicators were analyzed in one-way analysis of variance (ANOVA) procedure, followed by Tukey's multiple range test for post hoc comparisons (SPSS 20.0, IBM, USA). Microbiome, metabolite, protein expression, and immunofluorescence data were analyzed using two-tailed Wilcoxon rank-sum tests. Data are presented as least square means and standard error of the mean (SEM). *P* < 0.05 was considered as statistically significant.

## Results

### Lamb growth performance

Compared with CON, M-HMB significantly increased the final weight (*P* < 0.05) and average daily weight gain (ADG) of lambs (*P* < 0.05), while decreasing the feed conversion ratio (FCR; *P* < 0.05). In contrast, L-HMB, and H-HMB did not significantly affect the final weight, ADG, and FCR compared with CON. Additionally, no differences were found in the initial weight and average daily feed intake (ADFI) between the groups (Table 2).

**Table 2:** Growth performance of Tibetan lambs (*n* = 30)

Items^1^	Treatments^2^				SEM	*P*-value
	CON	L-HMB	M-HMB	H-HMB		
Initial weight, kg	16.71	16.88	16.62	16.62	0.072	0.532
Final weight, kg	37.19^c^	38.05^b^	38.96^a^	37.43^bc^	0.123	< 0.001
ADG, g/d	228.89^c^	234.90^bc^	247.38^a^	235.67^b^	1.036	< 0.001
ADFI, g/d	1761.73	1789.32	1,730.25	1754.06	9.695	0.193
FCR	7.70^a^	7.62^a^	7.01^b^	7.46^a^	0.054	< 0.001

^1^*ADG* Average daily gain, *ADFI* Average daily feed intake, *FCR* Feed conversion ratio, ADFI/ADG

^2^HMB was supplemented at 0 mg/kg (CON group), 430 mg/kg (L-HMB), 715 mg/kg (M-HMB), and 1,000 mg/kg (H-HMB) of diet

^a,b,c^Different letters represent significant differences (*P* < 0.05)

### Intestinal morphology, digestive enzyme and disaccharidase activities

**Fig. 1:**
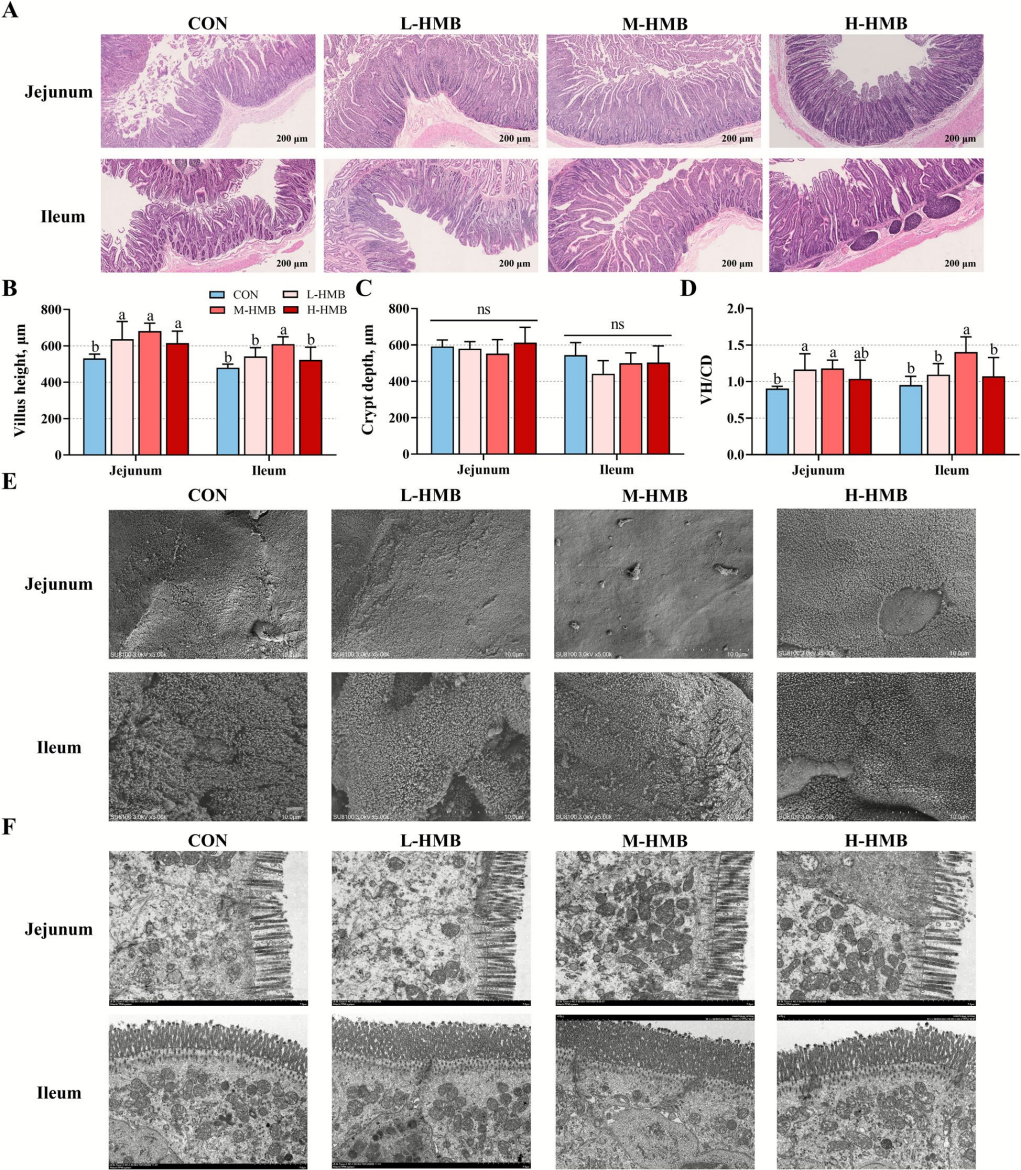
Effects of HMB on jejunum and ileum morphology in Tibetan lambs (*n* = 6). **A** HE stained section images. **B**–**D** The villus height, crypt depth, and the VH/CD ratio. **E** Scanning electron microscope images. **F** Transmission electron microscope images. HMB was supplemented at 0 mg/kg (CON group), 430 mg/kg (L-HMB), 715 mg/kg (M-HMB), and 1,000 mg/kg (H-HMB) of diet. Data in B, C, and D are represented as mean ± SEM. ^a,^^b^Different letters represent significant differences (*P* < 0.05)

**Fig. 2:**
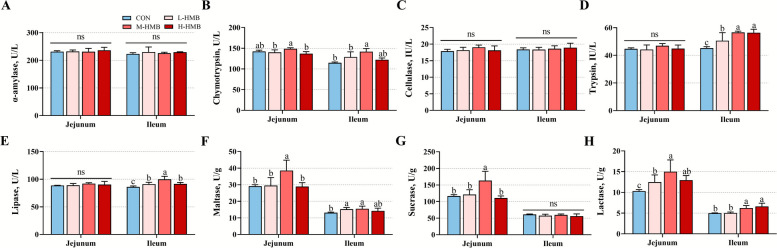
Effects of HMB on digestive enzyme and disaccharidase activities in jejunal and ileal contents of Tibetan lambs (*n* = 6). **A**–**E** Activities of digestive enzymes. **F–H** Activities of disaccharidases. HMB was supplemented at 0 mg/d (CON group), 430 mg/kg (L-HMB), 715 mg/kg (M-HMB), and 1,000 mg/kg (H-HMB) of diet. Data are represented as means ± SEM. ^a^^−c^Different letters represent significant differences (*P* < 0.05)

In the jejunum, L-HMB, M-HMB, and H-HMB significantly increased villus height (*P* < 0.05), while L-HMB and M-HMB also increased the VH/CD (*P* < 0.05) compared with CON. In the ileum, M-HMB significantly increased villus height (*P* < 0.05) and VH/CD (*P* < 0.05) compared with CON (Fig. 1A–D). Ultrastructural analysis by scanning electron microscopy and transmission electron microscopy confirmed enhanced small intestinal villus growth and architectural integrity in M-HMB (Fig. 1E and F). Compared to CON, M-HMB significantly enhanced the activities of chymotrypsin (*P* < 0.05), maltase (*P* < 0.05), sucrase (*P* < 0.05), and lactase (*P* < 0.05) in the jejunal content, and L-HMB and H-HMB also increased lactase activity (*P* < 0.05). In the ileal content, M-HMB significantly enhanced activities of chymotrypsin (*P* < 0.05), trypsin (*P* < 0.05), lipase (*P* < 0.05), maltase (*P* < 0.05), and lactase (*P* < 0.05) compared to CON (Fig. 2A–H).

### Anti-oxidative and immune parameters, and intestinal barrier-related gene expressions in jejunal and ileal tissues

**Fig. 3:**
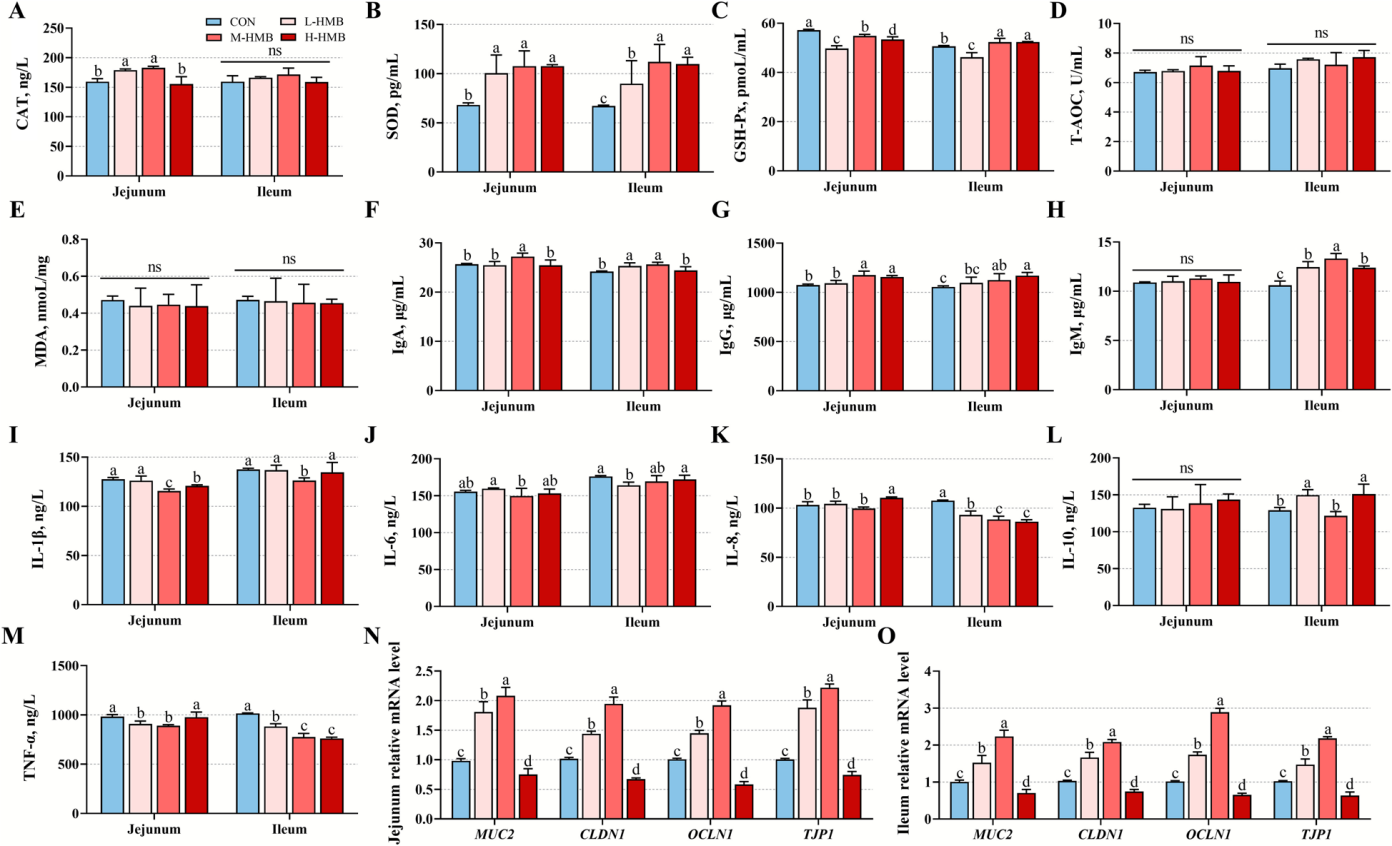
Effects of HMB on antioxidant parameters, immune cytokines expression, and intestinal barrier-related gene expression in the jejunum and ileum of Tibetan sheep (*n* = 6). **A**–**E** Activity of antioxidant enzymes and malondialdehyde concentration.** F–M** The expression of immune cytokines. **N**–**O** The expression of intestinal barrier-related genes. HMB was supplemented at 0 mg/d (CON group), 430 mg/kg (L-HMB), 715 mg/kg (M-HMB), and 1,000 mg/kg (H-HMB) of diet. CAT: catalase, GSH-Px: glutathione peroxidase, SOD: superoxide dismutase, T-AOC: total antioxidant capacity, MDA: malondialdehyde, IL-1β: interleukin-1 beta, IL-6: interleukin-6, IL-8: interleukin-8, IL-10: interleukin-10, TNF-α: tumor necrosis factor alpha. IgA: immunoglobulin A, IgG: immunoglobulin G, IgM: immunoglobulin M. Data are represented as means ± SEM. ^a^^−d^Different letters represent significant differences (*P* < 0.05)

Compared to CON, L-HMB and M-HMB significantly increased CAT activity (*P* < 0.05), while SOD activity (*P* < 0.05) was elevated by all HMB treatments (L-HMB, M-HMB, and H-HMB) in both the jejunum and ileum. Compared to CON, all HMB treatments decreased jejunal GSH-Px activity (*P* < 0.05), whereas ileal GSH-Px (*P* < 0.05) activity was reduced by L-HMB but increased by both M-HMB and H-HMB (Fig. 3A–E). M-HMB modulated immune responses compared to CON, increasing jejunal IgA (*P* < 0.05) and IgG (*P* < 0.05) concentrations while decreasing IL-1β (*P* < 0.05) and TNF-α (*P* < 0.05) concentrations. The effects were more pronounced in the ileum, with elevated IgA (*P* < 0.05), IgG (*P* < 0.05), and IgM (*P* < 0.05) concentrations and reduced IL-1β (*P* < 0.05), IL-8 (*P* < 0.05), IL-10 (*P* < 0.05), and TNF-α (*P* < 0.05) concentrations (Fig. 3F–M). Regarding intestinal barrier-related genes (*MUC2*, *CLDN1*, *OCEL1*, and *TJP1*), H-HMB decreased their expressions (*P* < 0.05), whereas L-HMB increased the expressions (*P* < 0.05) and M-HMB further elevated the expressions beyond those in L-HMB (*P* < 0.05) (Fig. 3N and O).

### Jejunal and ileal microbiota

**Fig. 4:**
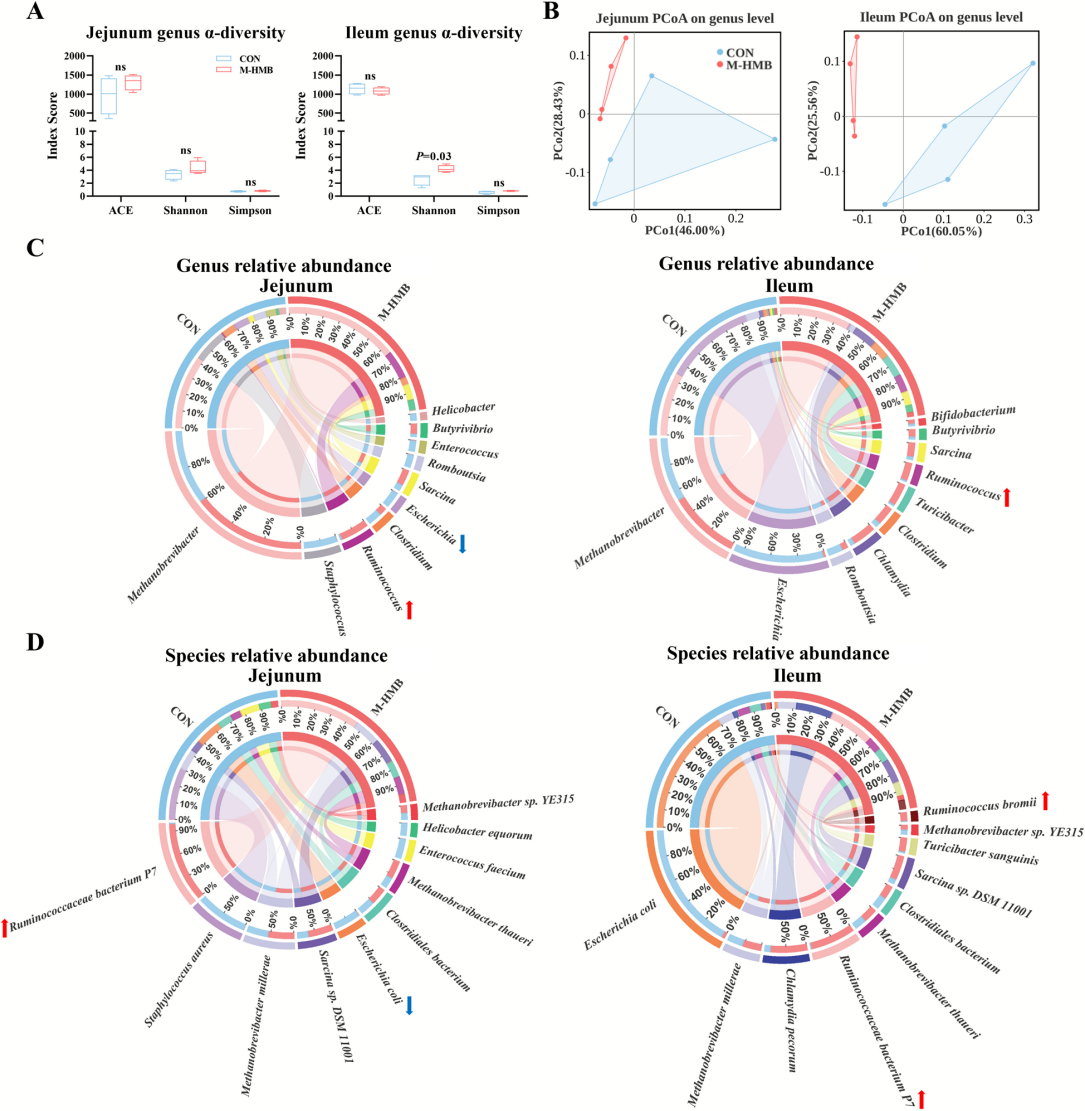
Effects of HMB on the diversity and microbial compositions in jejunal and ileal contents of Tibetan lambs (*n* = 4).** A** α diversity of microbial genera. **B** PCoA diagram of microbial genera. **C**–**D** Top 10 relative abundance of microbial genera and species. HMB was supplemented at 0 mg/kg (CON group), 430 mg/kg (L-HMB), 715 mg/kg (M-HMB), and 1,000 mg/kg (H-HMB) of diet. Data are represented as means ± SEM. Red arrows indicate relative abundance significant increases (*P* < 0.05) and blue arrows indicate relative abundance significant decreases (*P* < 0.05)

Preliminary results indicated that M-HMB supplementation produced the most pronounced improvements in growth performance, small intestinal morphology, digestive enzyme activity, antioxidant capacity, immune factors, and barrier function compared with L-HMB and H-HMB. To further investigate how M-HMB altered small intestinal fermentation, DNA was extracted from jejunal and ileal contents and subjected to metagenomic sequencing to examine changes in small intestinal microbiota. M-HMB had no significant effect on α-diversity in the jejunum (*P* > 0.05) but significantly increased the Shannon index in the ileum (*P* < 0.05) (Fig. 4A). Compared to the CON group, M-HMB treatment reshaped the microbial community composition in both the jejunum and ileum, as indicted by shifts in the relative abundances on the principal coordinates analysis (PCoA) plots (Fig. 4B).

**Fig. 5:**
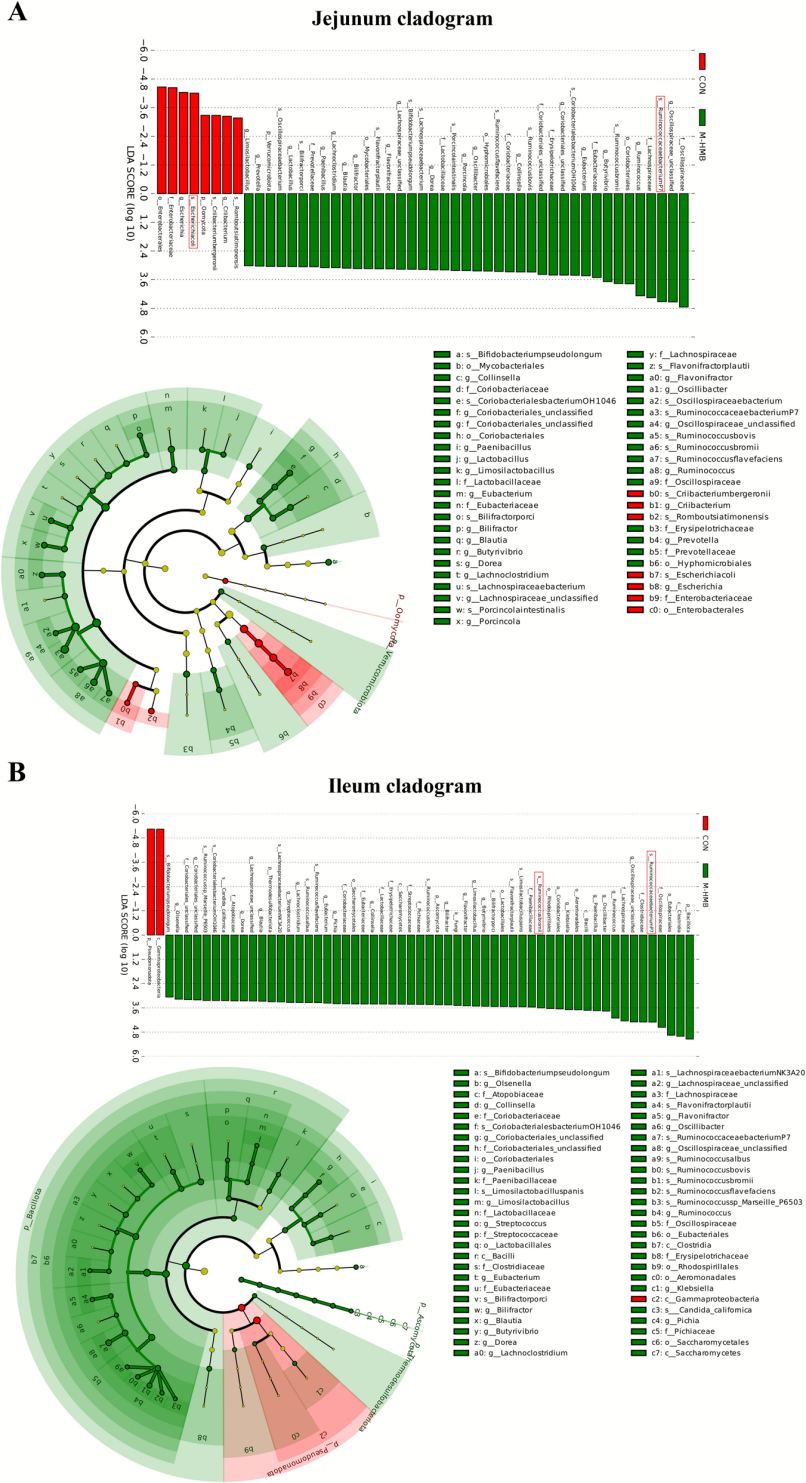
HMB altered the microbial community structure in jejunal and ileal contents of Tibetan lambs (*n* = 4). **A**–**B** LEfSe analysis diagram. HMB was supplemented at 0 mg/kg (CON group), 430 mg/kg (L-HMB), 715 mg/kg (M-HMB), and 1,000 mg/kg (H-HMB) of diet. LEfSe: linear discriminant analysis effect size

Among the relative abundances of the top 10 genera, M-HMB significantly increased relative abundance of *Ruminococcus* in both the jejunum and ileum (*P* < 0.05), and decreased *Escherichia* in the jejunum (*P* < 0.05; Fig. 4C). Among the relative abundances of the top 10 species, *Ruminococcus bacterium P7* was significantly increased in the jejunum (*P* < 0.05), while *Escherichia coli* was significantly decreased (*P* < 0.05). In the ileum, both *R. bacterium P7* (*P* < 0.05) and *R. bromii* (*P* < 0.05) were significantly increased (Fig. 4D). Linear discriminant analysis effect size (LEfSe) analysis further highlighted group-specific taxa. In the jejunum, *E. coli* had the highest species-level linear discriminant analysis (LDA) score in the CON, while *R. bacterium P7* exhibited the highest LDA score in M-HMB (Fig. 5A). In the ileum of the M-HMB, the top two species-level LDA scores belonged to *R. bacterium P7* and *R. bromii* (Fig. 5B).

### Microbial function changes in jejunal and ileal contents

**Fig. 6:**
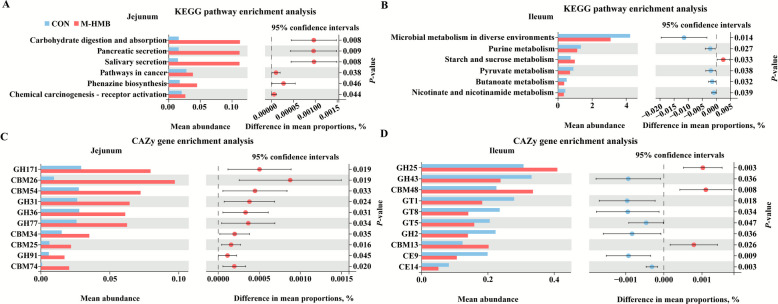
Effects of HMB on the pathway enrichment of microbes in jejunal and ileal contents of Tibetan lambs (*n* = 4) from the KEEG and CAZy databases. **A**–**B** KEGG pathway enrichment map. **C**–**D** CAZy gene enrichment map. HMB was supplemented at 0 mg/d (CON group), 430 mg/kg (L-HMB), 715 mg/kg (M-HMB), and 1,000 mg/kg (H-HMB) of diet. CBM: carbohydrate-binding modules, CE: carbohydrate esterase, GT: glycosyltransferase, GH: glycoside hydrolases

Microbial function changes in jejunal and ileal contents of both the CON and M-HMB lambs were analyzed based on KEGG database functional annotation of metagenomic data, as shown in Fig. 6A and B. Compared to the CON, the carbohydrate digestion and absorption pathways in the jejunum were significantly enhanced with M-HMB, and starch and sucrose metabolism were significantly enhanced with M-HMB (*P* < 0.05) in the ileum. Further CAZme analysis revealed that M-HMB intervention significantly (*P* < 0.05) increased the abundances of glycoside hydrolases (GH171, GH31, GH36, GH77, and GH91) and carbohydrate-binding modules (CBM26, CBM54, CBM34, CBM25, and CBM74) in the jejunum (Fig. 6C). Additionally, the increased levels of GH25, CBM48, and CBM13 were coupled with decreased (*P* < 0.05) abundances of GH43, glycosyltransferase (GT)1, GT8, GT5, GH2, carbohydrate esterase (CE)9, and CE14 (Fig. 6D). These findings demonstrated that M-HMB enhanced microbial carbohydrate digestion capacity and reduced carbohydrate synthesis capacity in the ileum.

### Metabolites in jejunal and ileal contents

**Fig. 7:**
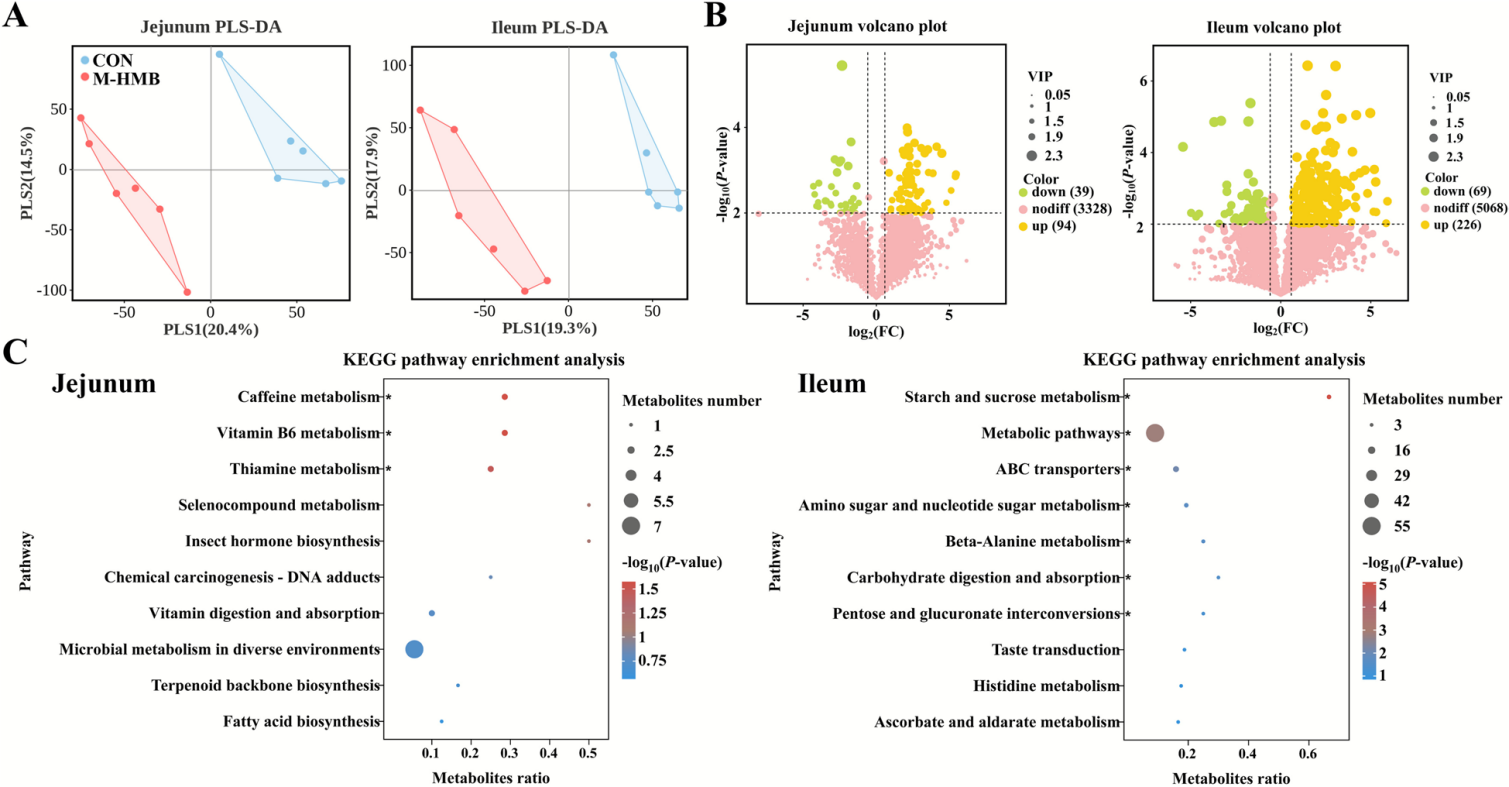
Effects of HMB on the metabolomics in jejunal and ileal contents of Tibetan lambs (*n* = 6). **A** PLS-DA diagram of total metabolites. **B** Volcano plot of total metabolite. **C**–**D** KEGG pathway enrichment diagram of differentially metabolized substances. HMB was supplemented at 0 mg/kg (CON group), 430 mg/kg (L-HMB), 715 mg/kg (M-HMB), and 1,000 mg/kg (H-HMB) of diet. ^*^P < 0.05

The PLS-DA score plots of the total metabolites in the intestinal contents revealed clean separation between the CON and M-HMB groups (Fig. 7A). A total of 133 and 295 differentially expressed metabolites were identified in the jejunum and ileum, respectively, of which 94 and 226 were upregulated (*P* < 0.05) (Fig. 7B). KEGG pathway enrichment analysis revealed that differential metabolites in the jejunum were significantly enriched in caffeine metabolism (*P* < 0.05), vitamin B6 metabolism (*P* < 0.05), and thiamine metabolism (*P* < 0.05) pathways. In the ileum, enriched pathways included starch and sucrose metabolism (*P* < 0.05), metabolic pathways (*P* < 0.05), ABC transporters (*P* < 0.05), amino sugar and nucleotide sugar metabolism (*P* < 0.05), beta-alanine metabolism (*P* < 0.05), carbohydrate digestion and absorption (*P* < 0.05), and pentose and glucuronate interconversions (*P* < 0.05) (Fig. 7C). These results demonstrated that M-HMB particularly influenced carbohydrate metabolisms in both the jejunum and ileum. SCFAs were quantified in the jejunal and ileal contents (Table 3). In the jejunum, the M-HMB exhibited significantly higher concentrations of total SCFAs (*P* < 0.05), acetate (*P* < 0.05), and propionate (*P* < 0.05), along with a lower acetate-to-propionate ratio (*P* < 0.05), compared to the CON group. In the ileum, M-HMB also significantly increased total SCFAs (*P* < 0.05), acetate (*P* < 0.05), and propionate (*P* < 0.05) concentrations (*P* < 0.05), but decreased the molar ratios of butyrate (*P* < 0.05) and valerate (*P* < 0.05).

**Table 3:** Effects of HMB on short-chain fatty acids (SCFAs) in jejunal and ileal contents of Tibetan lambs (*n* = 6)

Items	Treatments^1^				SEM	*P*-value
	CON	L-HMB	M-HMB	H-HMB		
Jejunum						
Total SCFAs, mmol/L	7.22^b^	7.89^b^	8.98^a^	7.70^b^	0.184	< 0.001
Acetate, mmol/L	5.81^b^	6.40^b^	7.16^a^	6.13^b^	0.169	0.007
Propionate, mmol/L	0.71^d^	0.83^c^	1.14^a^	0.94^b^	0.035	< 0.001
Butyrate, mmol/L	0.60	0.57	0.59	0.53	0.018	0.552
Valerate, mmol/L	0.10	0.09	0.09	0.10	0.006	0.704
Acetate/Propionate	8.26^a^	7.82^ab^	6.33^b^	6.55^b^	0.267	0.032
Acetate, %	80.44	80.93	79.51	79.49	0.482	0.774
Propionate, %	9.84^b^	10.63^ab^	12.87^a^	12.29^a^	0.393	0.031
Butyrate, %	8.30	7.27	6.55	6.84	0.266	0.082
Valerate, %	1.43	1.17	1.07	1.38	0.090	0.332
Ileum						
Total SCFAs, mmol/L	12.82^b^	13.94^b^	16.04^a^	14.03^b^	0.318	< 0.001
Acetate, mmol/L	9.83^b^	11.04^b^	12.66^a^	11.07^b^	0.285	< 0.001
Propionate, mmol/L	1.49^b^	1.51^b^	1.89^a^	1.47^b^	0.043	< 0.001
Butyrate, mmol/L	1.36	1.26	1.36	1.38	0.020	0.12
Valerate, mmol/L	0.13	0.13	0.13	0.12	0.001	0.17
Acetate/Propionate	6.58	7.32	6.72	7.59	0.170	0.10
Acetate, %	76.63^b^	79.05^a^	78.85^a^	78.86^a^	0.360	0.04
Propionate, %	11.70	10.94	11.81	10.46	0.222	0.089
Butyrate, %	10.65^a^	9.09^bc^	8.52^c^	9.81^ab^	0.217	< 0.001
Valerate, %	1.01^a^	0.91^b^	0.82^c^	0.87^bc^	0.020	< 0.001

*SCFAs* Short chain fatty acids

^1^HMB was supplemented at 0 mg/kg (CON group), 430 mg/kg (L-HMB), 715 mg/kg (M-HMB), and 1,000 mg/kg (H-HMB) of diet

^a^^−d^Different letters represent significant differences (*P* < 0.05)

### Relationships between the microbes, SCFAs, antioxidant and immune parameters, intestinal barrier-related gene expression, and lamb growth performance

**Fig. 8:**
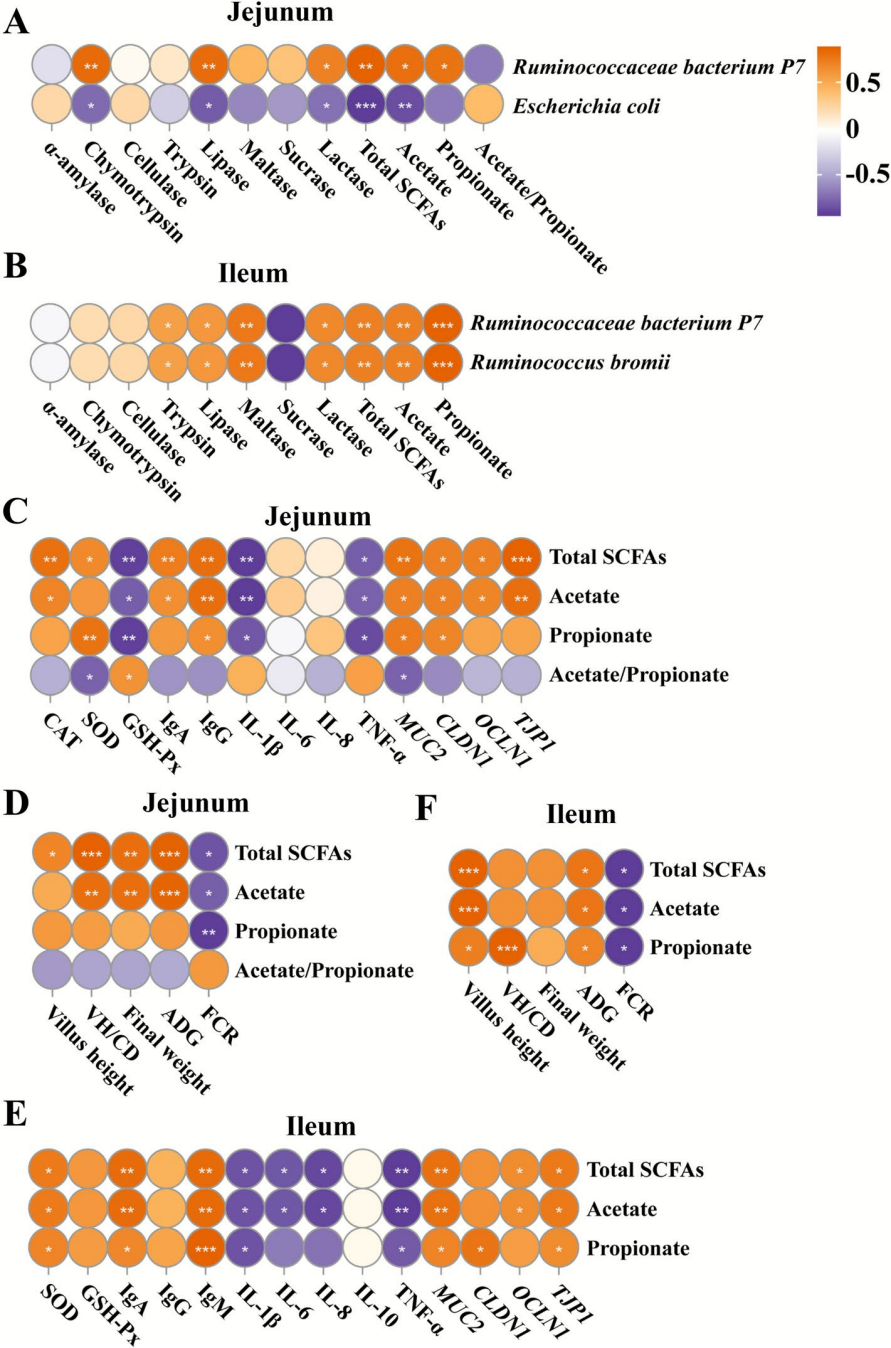
Significant Spearman correlations (*P* < 0.05) between intestinal microbiome, metabolites, SCFAs, antioxidant and immune parameters, intestinal barrier-related gene expression, and lamb growth performance (*n* = 4). **A**–**B** The correlations between microbiomes, digestive enzymes, disaccharidases, and SCFAs. **C**–**D** The correlations between SCFAs and antioxidant, immune parameters, intestinal barrier-related gene expressions, intestinal morphology, and lamb growth performance in the jejunum. **E**–**F** The correlations between SCFAs and antioxidant, immune parameters, intestinal barrier-related gene expressions, intestinal morphology, and lamb growth performance in the ileum. HMB was supplemented at 0 mg/kg (CON group), 430 mg/kg (L-HMB), 715 mg/kg (M-HMB), and 1,000 mg/kg (H-HMB) of diet. SCFAs: short-chain fatty acids, CAT: catalase, GSH-Px: glutathione peroxidase, SOD: superoxide dismutase, IgA: immunoglobulin A, IgG: immunoglobulin G, IgM: immunoglobulin M, IL-1β: interleukin-1 beta, IL-6: interleukin-6, IL-8: interleukin-8, IL-10: interleukin-10, TNF-α: tumor necrosis factor alpha, VH/CD: villi height/crypt depth, ADG: average daily gain, FCR: feed conversion ratio. ^*^P < 0.05; ^**^P < 0.01; ^***^P < 0.001

To investigate potential mediations of the intestinal differentially abundant microbes, Spearman correlations were examined between microbial abundances and digestive enzymes, disaccharidases, and SCFAs concentrations. In the jejunum, *R. bacterium P7* abundance was positively correlated (*P* < 0.05) with chymotrypsin, lipase, and lactase activities, and total SCFA and acetate concentrations, whereas *E. coli* abundance exhibited negative correlations (*P* < 0.05) with these parameters (Fig. 8A). In the ileum, both *R. bacterium P7* and *R. bromii* abundances were positively correlated (*P* < 0.05) with trypsin, lipase, maltase, and lactase activities, and total SCFAs, acetate, and propionate concentrations (Fig. 8B).

Significant correlations were also observed between SCFAs, antioxidant and immune parameters, and lamb growth performances. In the jejunum, total SCFAs, acetate, and propionate concentrations were positively correlated (*P* < 0.05) with CAT, SOD, IgA, IgG, and the barrier-related genes *MUC2*, *CLDN1*, *OCEL1*, and *TJP1*, as well as villus height, the VH/CD ratio, and lamb ADG, while negatively correlated (*P* < 0.05) with GSH-Px, IL-1β, and TNF-α levels, and with FCR (Fig. 8C and D). In the ileum, total SCFAs, acetate, and propionate concentrations were positively correlated (*P* < 0.05) with SOD, GSH-Px, IgA, IgM, *MUC2*, *CLDN1*, *OCEL1*, and *TJP1* levels, villus height, the VH/CD ratio, and lamb ADG, but negatively correlated (*P* < 0.05) with IL-1β, IL-6, IL-8, TNF-α, and FCR (Fig. 8E and F).

### HMB modulates the PLCβ/ERK signaling pathway in the ileum

**Fig. 9:**
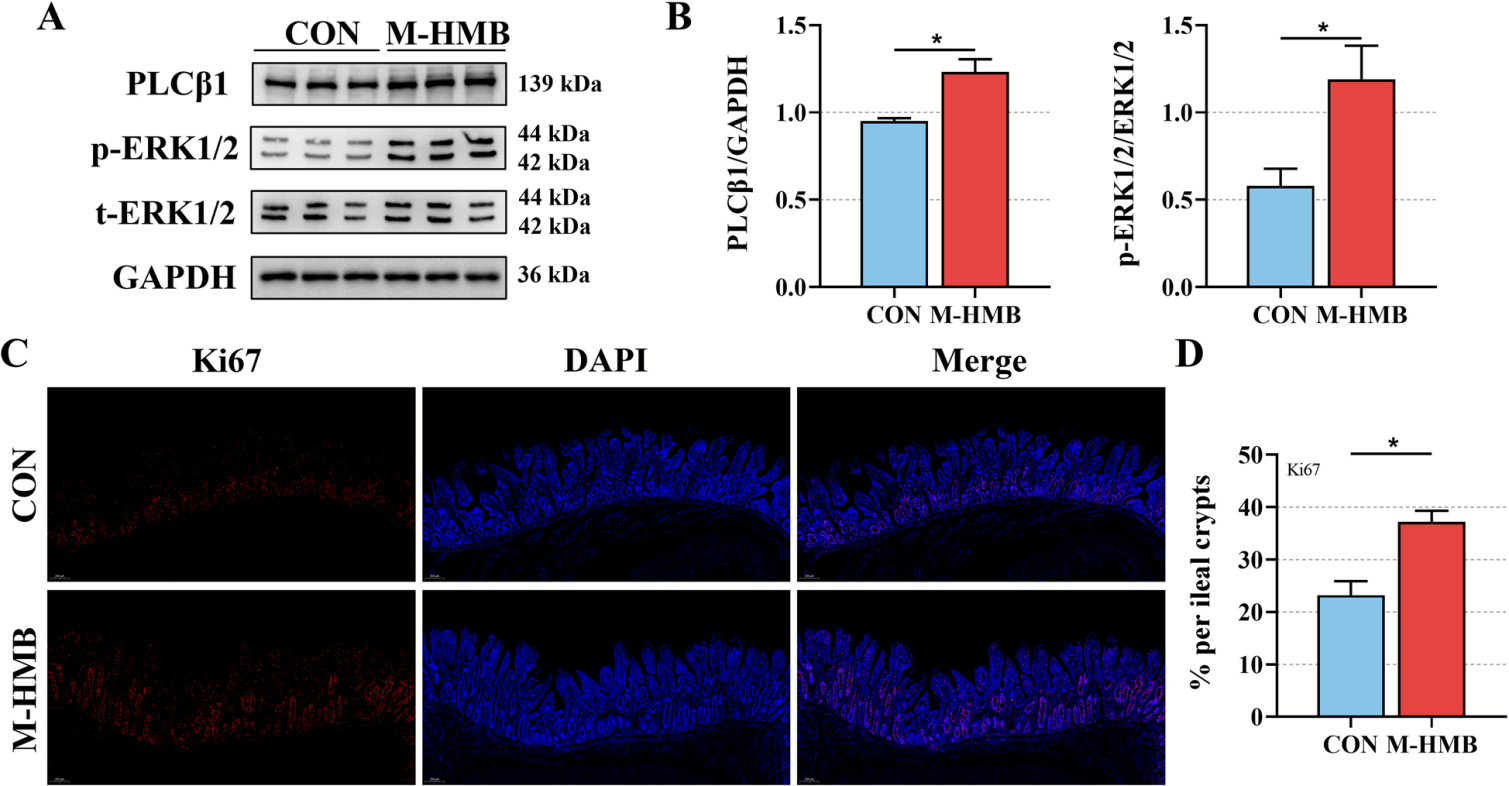
Effects of HMB on the PLCβ/ERK signaling pathway in the ileum of Tibetan lambs. **A**–**B** Representative protein blots of PLCβ1 and ERK1/2 (*n* = 3). **C**–**D** The proportion of Ki67-positive cells in the basal region of ileal crypts (*n* = 6). HMB was supplemented at 0 mg/kg (CON group) and 715 mg/kg (M-HMB) of diet. ERK1/2: extracellular signal-related kinase 1/2, PLCβ1: phospholipase C beta 1. Data are represented as means ± SEM. ^*^P < 0.05

Building on established evidence that HMB enhances SCFA production in Tibetan sheep small intestines and the documented phenotypic correlations of SCFAs, this study specifically investigated ileal tissue to elucidate HMB's. The potential regulatory effects of HMB on the PLCβ/ERK signaling pathway was examined in Western blot analysis. Protein expression levels of PLCβ1 and ERK1/2 were significantly higher (*P* < 0.05) in the M-HMB group compared to the CON group (Fig. 9A and B). Immunofluorescence staining further showed a marked increase (*P* < 0.05) in the proportion of Ki67-positive cells in the crypt basal regions following M-HMB treatment, indicating enhanced proliferative activity (Fig. 9C and D).

## Discussion

Tibetan sheep inhabiting the Qinghai-Tibet Plateau experience forage scarcity due to the region’s harsh environment, thus improving feed utilization efficiency can be a promising strategy to enhance animal productivity. In this study, we observed that dietary supplementation with 715 mg/kg HMB increased Tibetan lamb’s growth rate by 8% over three months without affecting feed intake, thereby improving feed efficiency. The improvement of feed efficiency and growth performance by HMB supplementation have been reported in other farming animal species, such as broiler chickens, pigs, and goats [17–19]. Collectively, these findings suggest that HMB appears to be a potential feed additive for livestock production.

The enhanced feed utilization efficiency with an appropriate level of HMB supplementation appeared to be associated with improved antioxidant capacity and immune homeostasis in the small intestine, contributing to strengthened intestinal barrier integrity in Tibetan lambs. Specifically, the M-HMB treatment enhanced CAT and SOD activities in the jejunum, and SOD and GSH-Px activities in the ileum. Our previous study showed that combined supplementation of resveratrol and HMB enhanced CAT and T-AOC activity and reduced MDA concentration in the rumen of Tibetan sheep [20]. The enhanced antioxidant capacity can reduce free radical-induced oxidative damage, compromise epithelial integrity and disrupt immune signaling, ultimately improving intestinal health [21, 22]. The association between HMB and immune functions has also been reported in fish, broilers, calves, and goats [23–26]. In pigs, maternal HMB supplementation during gestation improved the expression intestinal tight junction proteins in the offspring [7, 27]. These findings support our findings in the present study in Tibetan sheep. The increased villus height and VH/CD ratio, along with the elevated activities of digestive enzymes and disaccharidases observed in the M-HMB group, indicate an enhanced nutrient digestive and absorptive capacity in the intestine of Tibetan lambs. These improvements likely contributed to the improved feed efficiency and lamb growth performance. Increased villus height reflects an expanded absorptive surface area for nutrient uptake [28], while elevated activities of digestive enzymes and disaccharidases synergistically promote nutrient hydrolysis, thereby facilitating more efficient absorption [29]. Therefore, these findings demonstrate that M-HMB promotes intestinal maturation and improves nutrient digestion capacity in Tibetan sheep.

The small intestine harbors a diverse community of microorganisms and serves as a critical site for host–microbiota interactions. Microbial structure and metabolic activity in this region are be influenced by luminal conditions and closely associated with intestinal metabolite profiles [30]. In the present study, HMB treatment supplementation altered in the ileal microbial composition in Tibetan lambs. Specifically, compared to non-HMB supplementation, M-HMB increased the abundance of *R. bacterium P7* and *R. bromii* by 22-fold (2.281% vs. 0.100%) and 35-fold (0.843% vs. 0.023%), respectively, while decreased *E. coli* abundance by 99-fold (17.248% vs. 0.200%). *Ruminococcus* is a key anaerobic fermentative bacterium, predominantly colonizes in mammalian intestinal ecosystems including the rumen of ruminants and human colon. These bacteria decompose complex polysaccharides by secreting various carbohydrate-active enzymes, thereby producing SCFAs [31]. *R. bromii* is a well-known species within the *Ruminococcus* genus and plays an important role in degrading resistant starch in the intestine [32, 33]. The increase of these species in the intestine was reinforced by the corresponding enhancement of glycoside hydrolases (GH) and carbohydrate-binding modules (CBM) by CAZme analysis, as *Ruminococcus* is known to encode carbohydrate-active enzymes, particularly those in the GH family [31]. The enhanced carbohydrate degradation enzymes resulted in the increased SCFAs production, particularly acetate and propionate, in the small intestine. Collectively, these results suggest that dietary M-HMB supplementation might enhance carbohydrate degradation capacity in the intestine of Tibetan lambs. *E. coli* is a common intestinal bacterium, and although most strains are non-pathogenic, certain virulent types can cause disease [34]. In this study, we found a substantial decline of *E. coli* in the ileum of Tibetan lambs in response to the M-HMB treatment, suggesting that HMB might suppress the growth of this potential pathogen in Tibetan lambs.

SCFAs are vital mediator between the microbiota and host intestinal health [35]. Acetate and propionate function as both a crucial energy substrate for intestinal epithelial cells and direct modulators of host intestinal antioxidant, immunity, and barrier integrity [36, 37]. Research has demonstrated that subcutaneous acetate administration in rabbits significantly elevates cecal antioxidant capacity, as evidenced by increased activities of T-AOC, T-SOD, and GSH-Px [38]. In mice, oral administration of sodium acetate and sodium butyrate significantly decreased serum concentrations of IL-1β and TNF-α [39]. Propionate has been shown to enhance intestinal barrier function by upregulating tight junction protein expression while increasing mitochondrial superoxide dismutase activity, thereby mitigating oxidative stress [40–43]. In the present study, the M-HMB treatment increased the abundance of *Ruminococcus,* which was positively associated with SCFA concentration, while suppressed *E coli*, which showed a negatively association with SCFA concentration in the small intestine. The apparent significant associations between SCAFs and antioxidant capacity, immune parameters, and the expression of intestinal barrier-related genes based on Spearman correlation analysis underscore the critical roles of SCAFs in intestine function. SCFAs additionally activated the PLCβ/ERK signaling cascade through free fatty acid receptor 2 (FFAR2) stimulation, thereby modulating intestinal growth and developmental processes [44–47]. Furthermore, the elevated protein expression of PLCβ1 and ERK1/2, together with the increased Ki67-positive cell proportion, supported the promoting effect of HMB on intestinal epithelial proliferation.

This study has a few limitations. First, only male Tibetan lambs were included in the experiment. It is unclear whether interactions between HMB and sex of animals may influence the results. Second, the basal diet consisted of feed species commonly used in this region and contained approximately 30% of roughages, which is similar to dietary types used in farms. The diet contained 11.8% protein, lower than dietary protein levels typically used for fattening Tibetan lambs. HMB might function differently with diets containing high proportions of roughages or greater protein content. In addition, although growth performance was assessed using 30 lambs per group, only four lambs were used for metagenomic analysis. Despite the pronounced treatment effects, large individual variations were observed in this study, suggesting that more animals are required to achieve more robust characterization of microbial metagenomics. Thus, the findings of the present study should be validated in future with more tissue samples.

## Conclusions

In summary, supplementation with an appropriate level of HMB (715 mg/kg) improved the growth performance of Tibetan lambs. This improvement appeared to be associated with elevated *Ruminococcus* enrichment, which contributed to enhanced carbohydrate degradation and thereby to SCFA production in the small intestine. The shifts in microbial composition and SCFA concentrations were associated with greater antioxidant capacity, improved immune function, strengthened intestinal barrier integrity, and increased villus height. Collectively, these effects contributed to improved nutrient absorption and feed utilization efficiency in Tibetan lambs.

## Supplementary Information

Additional file 1: Fig. S1. Full uncropped Gels and Blots images.

Additional file 2: Table S1. Gene primers used for real-time quantitative PCR.

## Data Availability

Data of metagenomic sequencing have been deposited at Sequence Read Archive database: PRJNA1281845. These data are publicly available as of the date of publication.

## Abbreviations

ADG: Average daily gain
ADFI: Average daily feed intake
ANOVA: Analysis of variance
CAT: Catalase
CAZy: Carbohydrate-Active enZYmes Database
CBM: Carbohydrate-binding modules
CE: Carbohydrate esterase
CON: Control
ERK1/2: Extracellular signal-related kinase 1/2
FCR: Feed conversion ratio
GH: Glycoside hydrolases
GSH-Px: Glutathione peroxidase
GT: Glycosyltransferase
HMB: β-Hydroxy-β-methyl butyrate
IgA: Immunoglobulin A
IgG: Immunoglobulin G
IgM: Immunoglobulin M
IL-1β: Interleukin-1 beta
IL-6: Interleukin-6
IL-8: Interleukin-8
IL-10: Interleukin-10
KEGG: Kyoto Encyclopedia of Genes and Genomes
LDA: Linear discriminant analysis
LEfSe: Linear discriminant analysis effect size
MDA: Malondialdehyde
PCoA: Principal coordinates analysis
PLCβ1: Phospholipase C beta 1
PLS-DA: Partial least-squares discriminant analysis
SCFAs: Short-chain fatty acids
SOD: Superoxide dismutase
T-AOC: Total antioxidant capacity
TNF-α: Tumor necrosis factor alpha
VH/CD: Villi height/crypt depth
VIP: Variable importance projection
