# Supplementary figures and images for: Dietary β-hydroxy-β-methyl butyrate supplementation improves intestinal health and growth performance in Tibetan sheep lambs via modulating small intestinal microbiota
Source: J Anim Sci Biotechnol. 2026 Feb 9;17:25. doi: 10.1186/s40104-025-01345-z (PMC12884607; doi:10.1186/s40104-025-01345-z)

Figure S1 Full uncropped Gels and Blots images.

**PLCβ1**


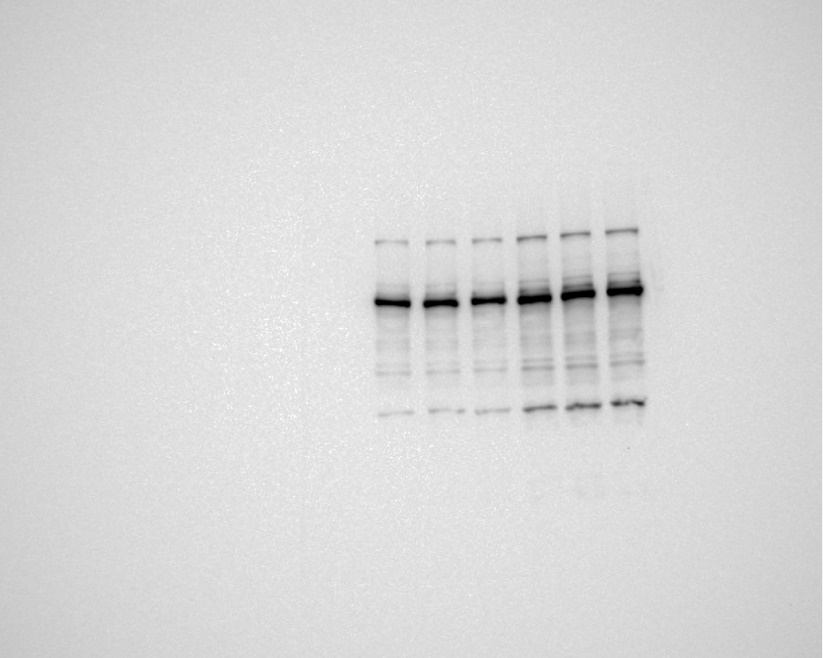


**p-ERK1/2**


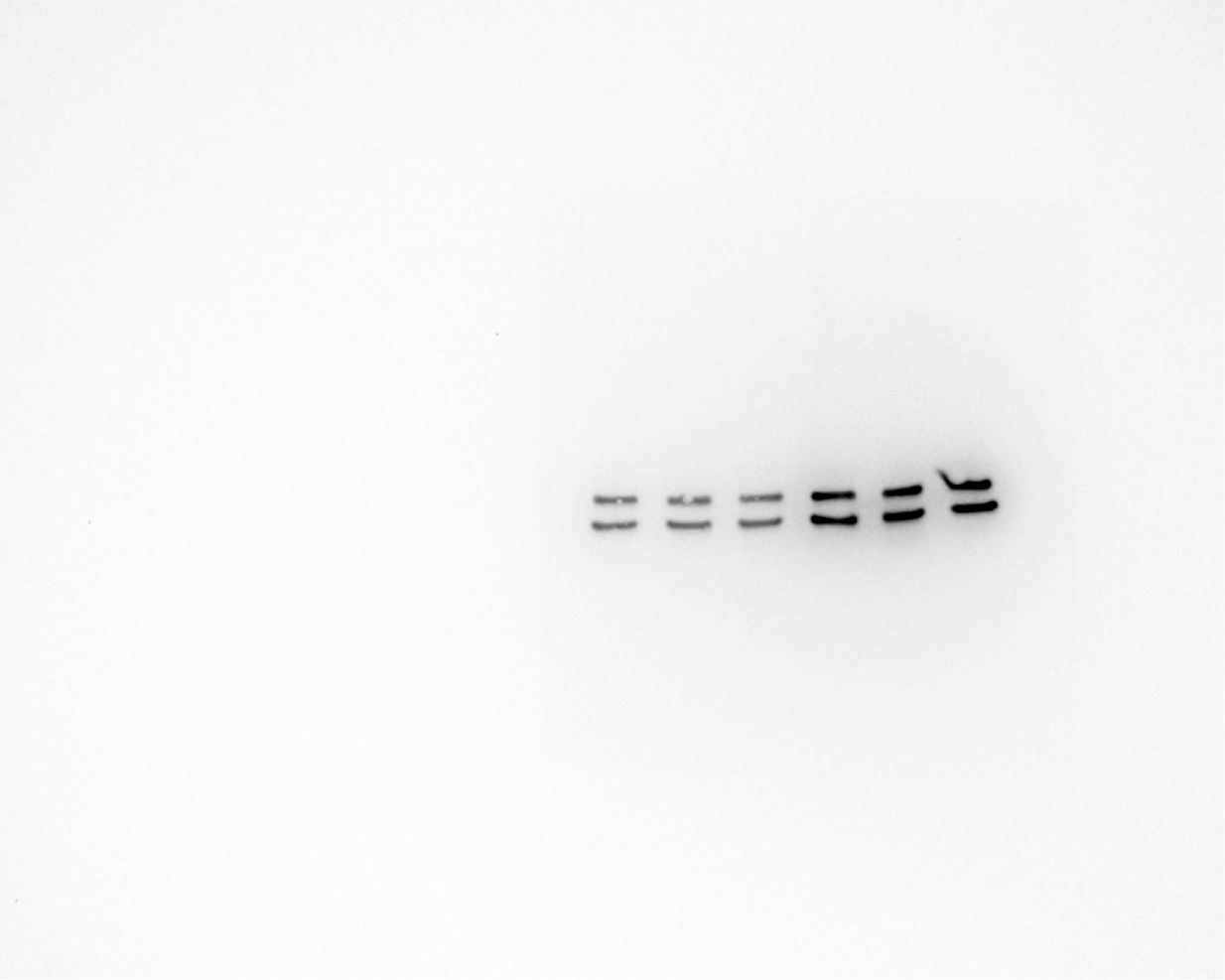


**t-ERK1/2**


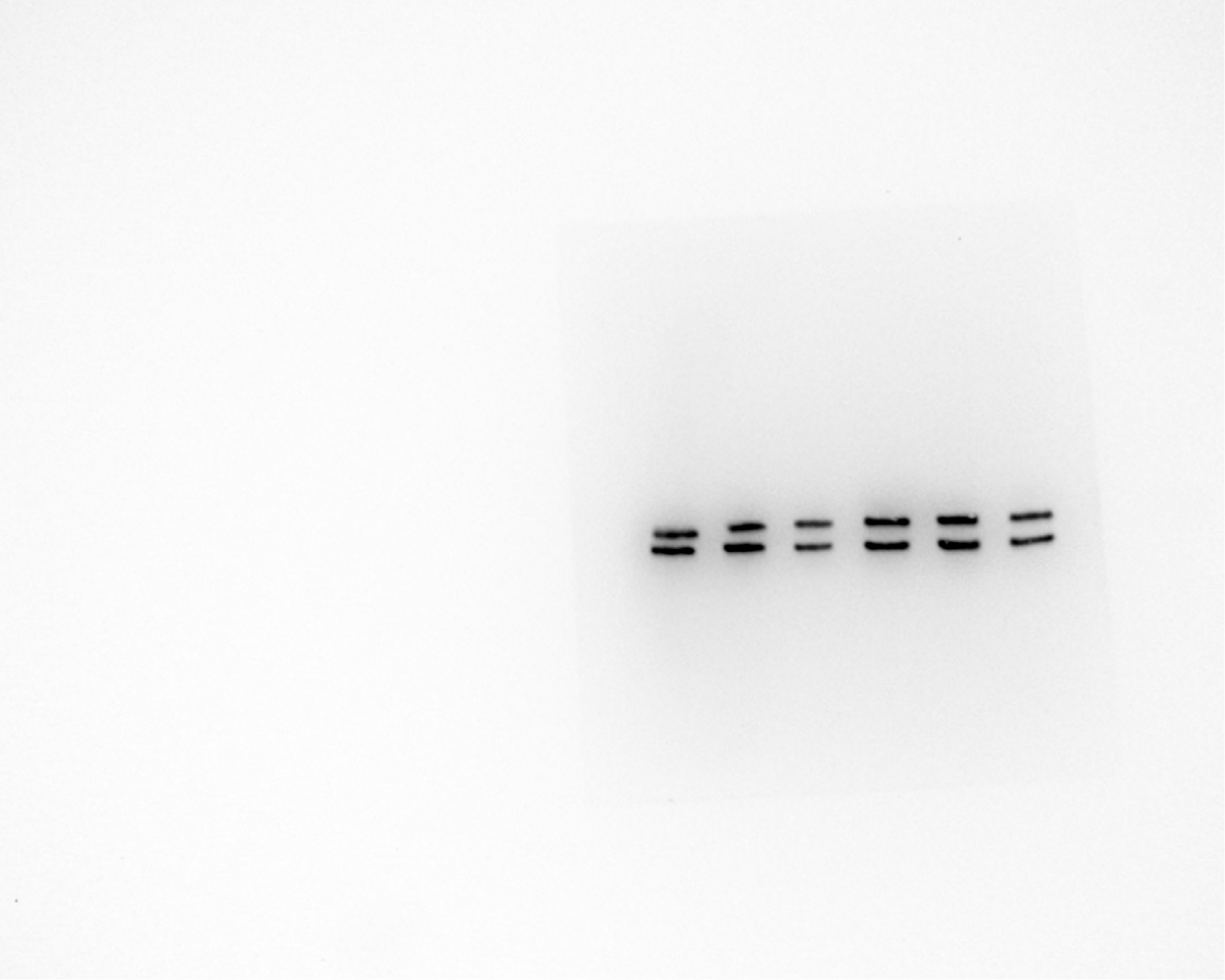


**GAPDH**


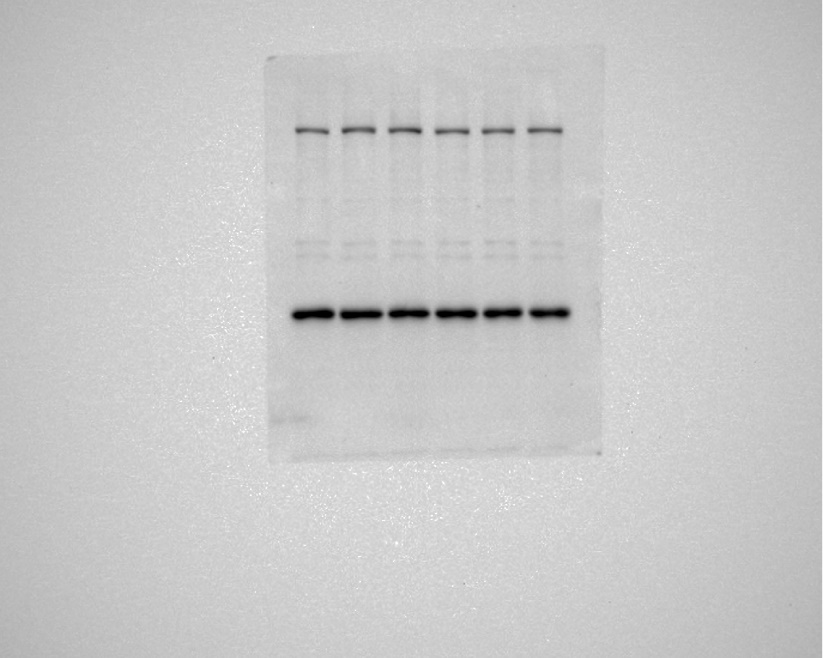

Supplement: Supplementary file 1 — Additional file 1: Fig. S1. Full uncropped Gels and Blots images. [file 40104_2025_1345_MOESM1_ESM.docx]
