# Supplementary material for: Dietary β-hydroxy-β-methyl butyrate supplementation improves intestinal health and growth performance in Tibetan sheep lambs via modulating small intestinal microbiota
Source: J Anim Sci Biotechnol. 2026 Feb 9;17:25. doi: 10.1186/s40104-025-01345-z (PMC12884607; doi:10.1186/s40104-025-01345-z)
Supplement: Supplementary file 2 — Additional file 2: Table S1. Gene primers used for real-time quantitative PCR. [file 40104_2025_1345_MOESM2_ESM.docx]

Table S1 Gene primers used for real-time quantitative PCR.

| Name | Primer sequence (5’-3’) | Tm (℃) | Size (bp) |
| --- | --- | --- | --- |
| *MUC2* | F-CCTCTGTGCTCACTGGGCTTCC | 65.7 | 148 |
|  | R-TCTGGCACTTGGATGAATAGACTGG | 64.6 |  |
| *CLDN1* | F-TTCAGGTCTGGCTGTTTTGGTTG | 64 | 203 |
|  | R-GTGTTGGGTAAGATGTTGTTTTCCG | 64 |  |
| *OCLN1* | F-TATGATGAGCAGCCTCCCAATGTG | 66 | 155 |
|  | R-GGATACGGTCGCTTCTCGTTCAC | 64.9 |  |
| *TJP1* | F-ATTCCAGACACTCTCCACAGCAGC | 64.6 | 198 |
|  | R-GGGCATACACAGGCTTTGGCTC | 65.6 |  |
| *β-actin* | F-CCTGCGGCATTCACGAAACTAC | 64.5 | 87 |
|  | R-ACAGCACCGTGTTGGCGTAGAG | 64.9 |  |
